# Cardiac output and CVP monitoring… to guide fluid removal

**DOI:** 10.1186/s13054-018-2016-y

**Published:** 2018-04-12

**Authors:** Matthieu Legrand, Sabri Soussi, François Depret

**Affiliations:** 1 AP-HP, GH St-Louis-Lariboisière, Department of Anesthesiology and Critical Care and Burn Unit, Paris, France; 2 University Paris Diderot, Paris, France; 3 UMR INSERM 942, Institut National de la Santé et de la Recherche Médicale (INSERM), Lariboisière hospital Univ Paris Diderot, F-75475 Paris, France

We read with interest the recently published position papers on central venous pressure (CVP) [1] and cardiac output (CO) [2] monitoring in critically ill patients and wish to further comment on their potential benefit. Haemodynamic monitoring is usually considered for haemodynamic support guidance during the early phase of shock. We would like, however, to emphasize the role of CVP and CO monitoring in another phase of critical illness, i.e. during volume depletion. Positive fluid balance and venous congestion have been associated with poor outcome in critically ill patients and in organ dysfunction (i.e. lung, kidney, liver, and gut) [3]. Volume depletion (i.e. using ultrafiltration or diuretics) is therefore a key part of the treatment of organ congestion and fluid overload after the initial phase of shock resuscitation. In these patients, the goal of volume depletion is to limit interstitial oedema and compartmental pressure [4]. Owing to the Starling forces, the intra-vascular hydrostatic pressure, oncotic pressure, and vascular permeability are the three factors driving trans-capillary filtration and therefore oedema generation. Volume depletion can limit trans-capillary filtration mostly through decreasing intra-venous and capillary hydrostatic pressure and possibly increasing intra-vascular oncotic pressure (Fig. 1). This strategy may, however, compromise venous return and therefore cardiac output, which may impact organ perfusion and organ function recovery. Initiation of volume depletion without haemodynamic monitoring may lead to either under- or over-treatment with a risk of hypoperfusion. The best balance would be to reach efficacy (i.e. decreasing intra-vascular hydrostatic pressure) without decreasing venous return and therefore stroke volume or cardiac output. Since venous return depends on the gradient between mean systemic pressure (Pms) and CVP, the ideal fluid depletion rate should probably aim at lowering Pms and CVP to the same extent (Fig. 2b), and therefore not comprising venous return. On the other hand, excessive or inappropriate fluid removal may induce a larger decrease in Pms than CVP and lead to a drop in venous return and stroke volume (Figs. 1 and 2b) [5]. As rightly pointed out by Monnet and Teboul, monitoring arterial pressure does not allow detection of all episodes of a decrease in cardiac output [2]. Guiding the rate of fluid volume depletion using haemodynamic monitoring allows monitoring both efficacy (i.e. lowering CVP) and tolerance (i.e. constant stroke volume). We strongly believe this approach may hasten organ function recovery, allow faster weaning from organ support, and therefore decrease the use of ICU resources.

**Fig. 1:**
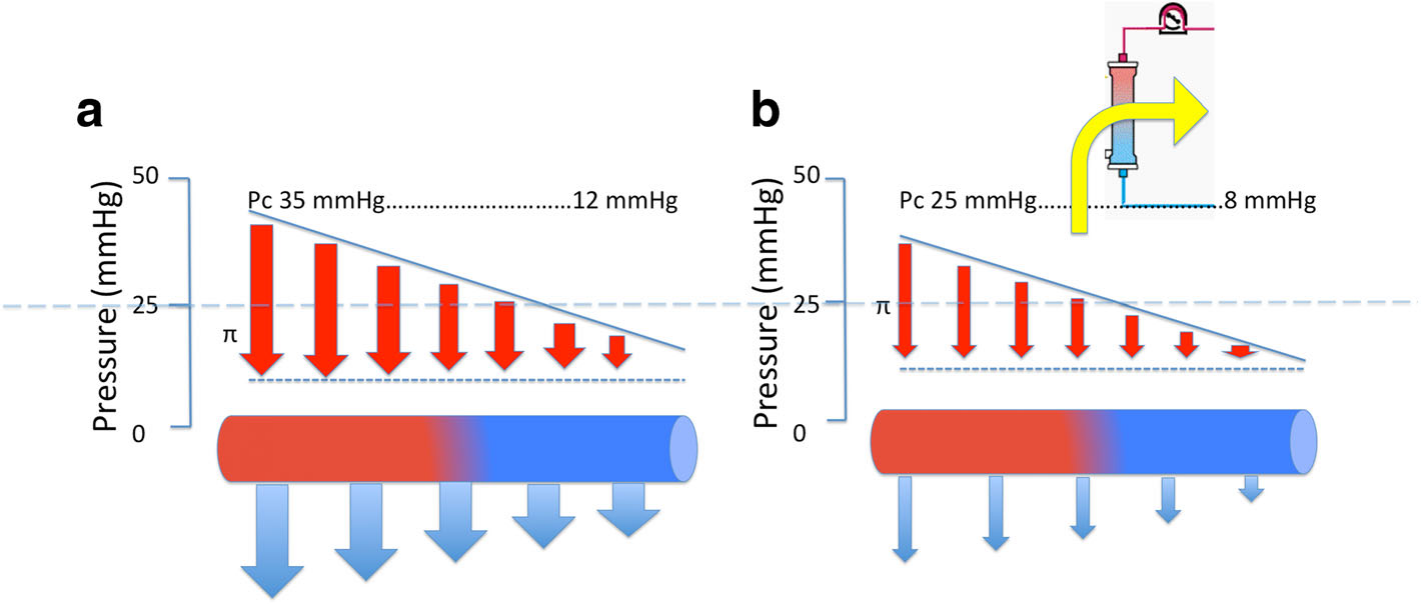
Theoretically, owing to Starling forces, volume depletion should lead to a decrease in intra-vascular hydrostatic pressure (Hp; from panel **a** to panel **b**) together with an increase in oncotic pressure (π), therefore decreasing the trans-capillary filtration rate and interstitial oedema generation

**Fig. 2:**
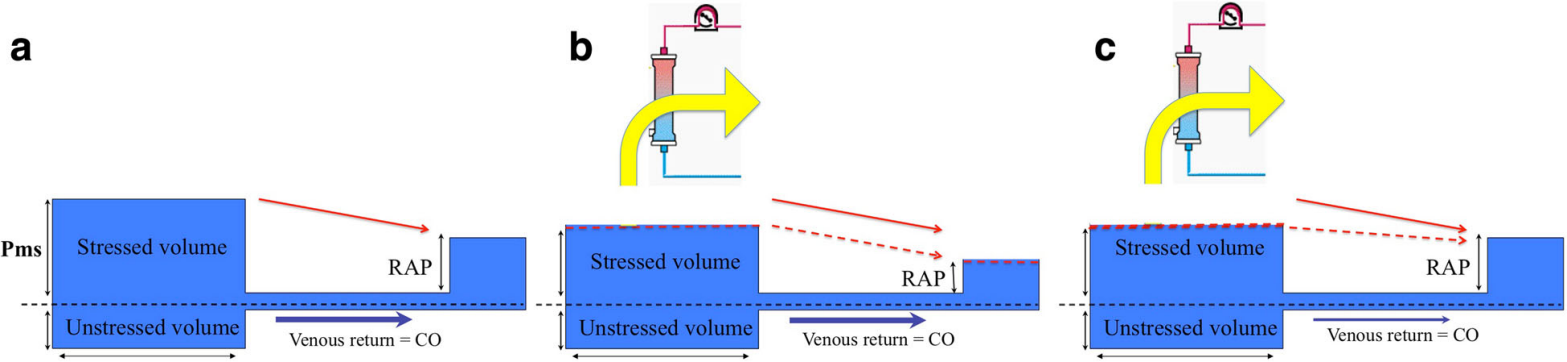
Hemodynamic monitoring during fluid depletion allows the assessment of both efficacy (i.e. decrease in intravascular pressure through central venous pressure (CVP) monitoring) and tolerance through stable stroke volume. Fluid depletion (from **a** to **b**) should indeed aimed at decreasing intravascular venous pressure without compromising the gradient between mean systemic pressure (Pms) and right atrial pressure (RAP) or CVP, and therefore maintaining venous return and cardiac output (CO). Excessive or inappropriate fluid removal may lead to a higher decrease of Pms than CVP, therefore compromising venous return and CO (**c**)

## Abbreviations

CO: Cardiac output
CVP: Central venous pressure
Pms: Mean systemic pressure
